# Editorial: Vascular injury in systemic diseases: current concepts and future perspectives

**DOI:** 10.3389/fcvm.2025.1656542

**Published:** 2025-07-21

**Authors:** Allegra Battistoni, Theodoros Dimitroulas, Panagiota Anyfanti

**Affiliations:** ^1^ Clinical and Molecular Medicine Department, Sapienza University of Rome, Rome, Italy; ^2^ Fourth Department of Internal Medicine, Hippokration Hospital, Thessaloniki, Greece; ^3^ 3rd Department of Internal Medicine, Papageorgiou Hospital, Aristotle University of Thessaloniki, Thessaloniki, Greece

**Keywords:** vascular injury, vasculature, systemic diseases, cardiovascular system, endothelial dysfunction

**Editorial on the Research Topic**
Vascular injury in systemic diseases: current concepts and future perspectives

**Figure 1:**
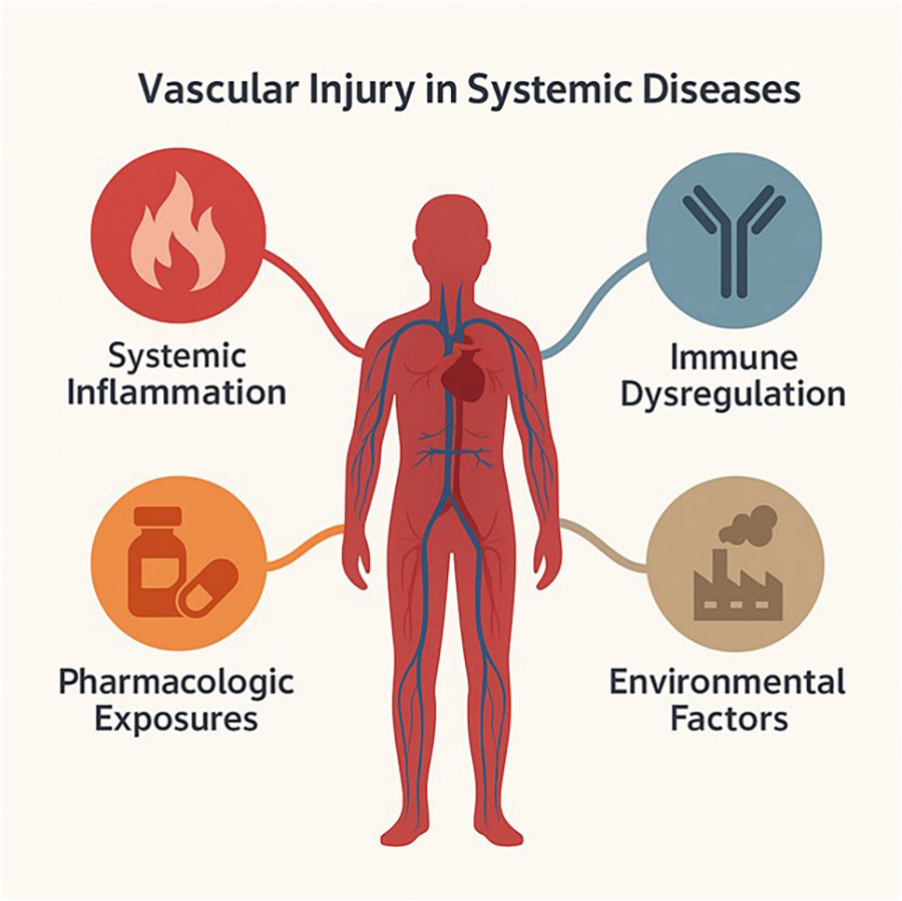
Complex and diverse factors interact and contribute to vascular injury in systemic diseases, including immune activation, metabolic dysregulation, chronic inflammation, pharmacological exposures, and/or environmental stressors.

The vasculature is no longer viewed simply as a passive conduit for the circulation of blood, but rather as a dynamic and highly responsive system that reflects and shapes the status of systemic health (1). In recent years, vascular injury has emerged not merely as a downstream consequence of chronic disease but as a crucial driver of multi-organ pathology (2, 3). Whether through immune activation, metabolic dysregulation, chronic inflammation, or environmental stressors, systemic diseases frequently converge on the vascular system, compromising its integrity and function at various levels and with diverse clinical consequences (Figure 1).

The articles collected in this Research Topic, *Vascular Injury in Systemic Diseases*, illuminate how this convergence manifests in both common and rare scenarios, and how the cardiovascular system, whether at the level of capillaries, major vessels, or the heart itself responds to these multifaceted challenges.

One major contribution of this collection is the emphasis on the complexity of vascular remodeling in chronic systemic diseases. For example, systemic sclerosis and cirrhotic portal hypertension, both classically studied for their fibrotic or hemodynamic component, were here reframed in vascular terms. In the contribution by Heilmeier et al. on the systemic sclerosis, low-grade inflammation and persistent immune activation seem to promote atherogenesis through chronic endothelial stress, challenging clinicians to recognize the vascular burden in what is often mischaracterized as purely connective tissue disease (Heilmeier et al.). Similarly, the pathophysiology of portal hypertension is enriched by a focus on intra- and extrahepatic vascular dysregulation, offering new perspectives on the interplay between liver disease and systemic vascular remodeling in the paper by Li et al.

Genetic approaches further deepen this perspective. Indeed, Mendelian randomization analysis can provide a powerful tool in addressing the limitations of observational studies and reveal causal effects. A Mendelian randomization study by Deng et al. included in this Topic explores the vascular implications of insomnia, an increasingly prevalent condition often overlooked as a cardiovascular risk factor. By demonstrating that genetically predicted insomnia increases the risk of myocardial infarction through modifiable intermediaries such as smoking and body mass index, the authors highlight the need to expand our understanding of behavioral contributors to vascular injury (Deng et al.). Another study by Lin et al. aims to fill the gap in research concerning the relationship between lupus and cardiac structure and function using similar methodology. The conclusion, supporting a causal relationship between lupus traits and alterations in cardiac structure and function, brings fresh insight into how autoimmune diseases may exert subclinical but progressive cardiovascular damage (Lin et al.).

The role of infection and immune activation in provoking vascular injury is also a recurring theme. Huang et al. evaluate the emergence of anti-neutrophil cytoplasmic antibodies following SARS-CoV-2 infection, pointing to the virus's capacity to induce autoimmune vascular responses even in the absence of overt vasculitis. Though the pandemic's acute effects may be receding, its immunological sequelae remain a fertile ground for understanding vascular reactivity in post-infectious contexts (Huang et al.).

Several clinical case reports in this Topic serve as reminders of how vascular involvement may dominate or complicate systemic illnesses. Teng et al. striking example details a case of granulomatosis with polyangiitis mimicking a mass in the aortic root, a rare but instructive instance of how large-vessel vasculitis can masquerade as neoplastic disease (Teng et al.). In such cases, misdiagnosis can carry fatal consequences, underlining the importance of maintaining a high index of suspicion for immune-mediated vascular pathology.

Other cases document severe, therapy-related vascular and hematological complications, including heparin-induced thrombocytopenia after plasmapheresis by Zhou et al. and propylthiouracil-induced ANCA-associated vasculitis complicated by granulocytopenia and hemophagocytosis by Chen et al. These reports reveal how therapeutic interventions, especially in immunologically fragile individuals, may act as unintentional vascular stressors.

Even within the pediatric population, the consequences of microvascular injury might be severe. A rare but illuminating case of livedoid vasculopathy in children is presented by Qu et al., underscoring how microcirculatory disorders, though often underdiagnosed, can lead to painful and debilitating outcomes (Qu et al.). Such contributions expand the conversation around vascular injury beyond adult cardiovascular risk, into realms where early recognition may alter long-term prognosis.

What ties these studies together is not a single anatomical structure or disease mechanism, but a shared recognition that vascular injury is a central, actionable pathway through which systemic diseases evolve and express their severity. The endothelium, the vessel wall, the cardiac muscle, and the immune interface all represent sites where systemic disease can either be intercepted or exacerbated. This requires a shift in both research and clinical practice from viewing vascular damage as a late-stage complication to treating it as a primary target for prevention and intervention.

We hope that this collection of articles will serve not only as a scientific contribution but also as a call to shift our clinical and research focus toward the vasculature—not simply as a passive conduit but as a dynamic, vulnerable, and actionable player in systemic disease. By placing vascular injury at the center of multidisciplinary discourse, we may unlock new strategies to predict, prevent, and treat the complications of systemic illness.
